# Survey of Afterschool Programs Suggests Most Offer Fruit and Vegetables Daily

**DOI:** 10.5888/pcd15.170396

**Published:** 2018-05-31

**Authors:** Jean L. Wiecha, Pamela A. Williams, Kristen C. Giombi, Amanda Richer, Georgia Hall

**Affiliations:** 1 RTI International, Waltham, Massachusetts; 2 RTI International, Research Triangle Park, North Carolina; 3 Wellesley College, Wellesley, Massachusetts

## Abstract

**Introduction:**

Most children underconsume fruit and vegetables. This study estimated the frequency and quality of fruit and vegetables offered during snack in US afterschool programs and examined program-level factors associated with offering them, including awareness and use of the National AfterSchool Association Healthy Eating and Physical Activity standards.

**Methods:**

We conducted descriptive analyses and regression modeling by using data collected from 684 National AfterSchool Association members and their colleagues via a 2015 online survey.

**Results:**

At the previous snack, 63% of respondents offered fruit, a vegetable, or both, with 42% offering only fruit, 18% offering fruit and vegetables, and 3% offering only vegetables. The quality of the items offered showed that most respondents selected the healthiest options, such as fresh fruit and vegetables. Controlling for other factors, we found that factors independently associated with offering fruit, vegetables, or both were membership in the National AfterSchool Association, using the standards for menu planning, and training staff members in healthy eating more than once a year. Programs run by school districts were less likely to offer fruit than programs run by other organizations.

**Conclusion:**

Membership in the National AfterSchool Association and use of its Healthy Eating and Physical Activity standards are associated with offering fruit and vegetables during snack at afterschool programs staffed by National AfterSchool Association members and their colleagues across the United States. With over a third of sites surveyed offering neither a fruit nor a vegetable at the previous snack, additional implementation of the standards is still needed.

## Introduction

Consuming fruit and vegetables helps children achieve appropriate intake of underconsumed nutrients, reduces the risk of developing chronic diseases, and helps children manage their weight (1). The US Department of Agriculture (USDA) recommends that school-age children consume 1.5 to 2 cups of fruit and 1.5 to 3 cups of vegetables per day depending on age and sex (2). Nonetheless, compared with Healthy People 2020 targets, US children eat about two-thirds of the recommended amount of fruit and less than half the recommended amount of vegetables daily (3). Overall, 6 of 10 US children underconsume fruit and more than 9 of 10 children underconsume vegetables (4).

More than 10 million US children participated in afterschool programs in 2014, almost half from low-income households (5). Because they reach so many children in need and typically offer food daily, afterschool programs can have a positive impact on children’s diets and improve equity in access to healthy food (6). Currently, several regulatory and advisory mechanisms influence food served in afterschool programs. For example, afterschool programs may participate in USDA child nutrition programs such as the Child and Adult Care Food Program (CACFP) and the National School Lunch Program (NSLP), both of which have menu pattern guidelines for snacks and meals. CACFP and NSLP allow, but do not require, afterschool providers to serve fruit and vegetables daily. Afterschool programs may also participate in the Summer Food Service Program, which requires that all meals include 2 servings of fruit, vegetables, or both (7), but participation is seasonal. Apart from these federal programs, afterschool programs may face nutrition requirements imposed by local jurisdictional rules such as licensing regulations, although licensing language varies widely (8). Outside of regulation, afterschool menus may reflect organization-specific policies and initiatives. For example, many large afterschool providers have nutrition initiatives grounded in the 2011 National AfterSchool Association Healthy Eating and Physical Activity (NAA HEPA) standards (6).

Although regulatory and advisory mechanisms are helpful, Story et al recommended in 2008 that studies assess the nutritional quality of snack foods and beverages served in afterschool programs, yet national-level data remain scarce (9). A 2012 study of NSLP afterschool snack menus showed that in school year 2009–2010, only 17% of menus included fruit and only 2% included a vegetable (10). In a recent South Carolina study, about 25% of afterschool programs served fruit and vegetables daily at baseline (11). This improved significantly upon intervention (12), joining older studies showing that intentional efforts can improve snack quality in afterschool programs (9,13–16). Although the NAA HEPA standards have been widely disseminated and adopted, their impact on afterschool snack quality is unknown. Additional national information on afterschool snacks would provide a benchmark for future progress and inform policy development and training and technical assistance needs.

We conducted a national survey of NAA members affiliated with afterschool programs throughout the United States to estimate the frequency of offering fruit and vegetables during afternoon snacks and their quality. We examined whether certain factors were independently associated with offering fruit, vegetables, or both, including awareness and use of the NAA HEPA standards, staff training on healthy menu development, participation in federal child nutrition programs, and organizational affiliation.

## Methods

### Participants

Survey participants were from NAA’s August 2015 membership database of 7,953 records (used with permission). Our goal was to survey one NAA member from each afterschool service provider in the United States. To reach this goal, we eliminated 31 records with addresses outside the United States, 1,289 that did not include an organization name, and 983 that were not service providers (eg, university faculty, public agency staff). Next, among the remaining 5,650 records, we identified 3,677 unique organization names. We randomly selected one record to keep when multiple records listed the same organization name, eliminating 2,005 records. We emailed invitations to the remaining 3,645 members and, after eliminating 214 undeliverable addresses, arrived at our final recruitment sample of 3,431 NAA members.

### Instruments

We developed an online survey that obtained descriptive information and assessed nutrition practices consistent with the NAA healthy eating (HE) standards. Five items assessed staff training for healthy eating, and a 17-item food and beverage checklist obtained reports of foods served “yesterday” or on the previous program day if yesterday was a weekend or holiday. The checklist asked about food and beverage items and their qualities (eg, if fruit was fresh, frozen, or canned) and drew on NAA standard HE-01 (Box), the Alliance for a Healthier Generation’s HOST Initiative (17), and USDA Smart Snacks guidelines (2). We pretested the survey at 4 afterschool programs, revising until pretesters offered no further suggestions. The pretest used a cognitive testing protocol (18) to ensure that respondents understood the questions, did not perceive bias in wording, felt response categories were appropriate, and felt they could answer the questions accurately.

**Box. Healthy Eating Standards Adopted by the National Afterschool Association, April 2011**

The National AfterSchool Association adopted 6 healthy eating (HE) standards and 5 physical activity standards in April 2011 (http://naaweb.org/images/NAA_HEPA_Standards_new_look_2015.pdf). Shown are the 6 HE standards that address food and beverage quality and infrastructure supports that include staff training, nutrition education, social support, program support, and environmental support.

**HE 01. Content and Quality**

Programs serve foods and beverages in amounts and types that promote lifelong health and help prevent chronic disease. These include minimally processed foods made with whole grains and heart-healthy fats or oils and without added sugar or trans fats; fruits and vegetables; and beverages made without added sugars.

**HE 02. Staff Training **

Staff members regularly participate in learning about healthy eating grounded in effective training models using content that is evidence-based.

**HE 03. Nutrition Education Curriculum**

Programs that offer nutrition education classes will ensure that materials presented to children are evidence-based, do not support a particular industry or food sector agenda, and are delivered by qualified personnel.

**HE 04. Social Support**

The program creates a social environment, including positive relationships, that encourages children to enjoy healthy foods. Research shows that children’s food choices are influenced not only by food appearance, taste, and familiarity, but also by social factors including peers, role models, group dynamics, and having healthy options.

**HE 05. Program Support**

Infrastructure supports healthy eating through management and budgeting practices.

**HE 06. Environmental Support**

The program’s physical environment supports healthy eating. Availability of vending machines, advertising and availability of kitchen facilities can all influence food choices and food availability.

### Procedure

Wellesley College’s institutional review board determined that this study was exempt from human subjects’ research requirements. We administered the final survey through SurveyMonkey from September 23 through October 26, 2015. We emailed invitations, allowing recipients unable to complete the survey to invite a more qualified person within their organization. If they had responsibilities at multiple afterschool sites, respondents were instructed to select one and focus on it when responding to food, beverage, and training questions.

We received 789 responses. We eliminated 36 that were substantially incomplete. We also eliminated 24 responses from organizations that had 2 respondents; for these, we kept the more complete response (n=17) or the response from the more senior respondent when both were complete (n=7). The final analyzable data set thus included 729 responses (21% of 3,431 invitees). We analyzed data from the 684 (94% of 729) respondents who reported sometimes or always serving an afternoon snack. Responses came from 49 states. Four randomly selected respondents received a $75 incentive.

#### Independent variables

We examined a range of independent variables that could affect whether fruit and vegetables are served as snacks. Indicator variables describing the afterschool program included whether a site was a 21st Century Community Learning Center (21st CCLC), which are afterschool programs run by school districts and focusing on academic enrichment for high-risk, low-income populations; was accredited (accreditation is managed through the Council on Accreditation (19) and is a voluntary process with some of the current standards reflecting the NAA HE Standards); participated in CACFP and NSLP (described previously), because the NAA Standard HE-05 Program Support specifically recommends that afterschool programs participate in federal food programs when possible; and whether a site was operated by a school district versus any other response (eg, YMCA, Boys & Girls Clubs, Parks and Recreation, faith-based, write-in). We also assessed whether a site was licensed. Licensing regulations are typically administered through a state agency, vary widely, and may include nutrition standards in their regulations; although they are not a specific indicator of nutrition quality, they may be considered a proxy for identifying sites that achieved capacity to meet quality standards. For many of these variables, we provided 3 response options (yes, no, don’t know), which we dichotomized as yes versus no/don’t know for regression analyses. We grouped don’t know responses with no to be conservative and because the affirmative condition would likely be obvious to program staff.

We also included variables that assessed NAA influence: current respondent membership in NAA, respondent awareness of the NAA HE standards before participating in the survey, and level of familiarity with the standards. We also asked whether the respondent’s site currently used one or more of the standards to guide “how they plan and serve foods and beverages.” These variables had categorical response options that we dichotomized for model building.

Because staff training on healthy eating is addressed in NAA Standard HE-02 (6) and could influence whether fruit and vegetables are served, we included 2 variables describing training frequency for site staff members not involved in menu planning (received training once a year; received training more than once a year). Both training variables were set to 1 if yes and 0 for all others, including missing values.

#### Dependent variables

Our dependent variables for this analysis came from 2 items asking if sites had offered fruit or vegetables during afternoon snack on the previous program day, not including juices. Follow-up questions assessed item qualities, such as whether the fruit or vegetable was fresh, frozen, or canned, and, to ascertain nutritional variety, if the vegetables were green versus red, yellow, or orange. We derived 3 binary (0/1) outcome measures for our models: 1) offered a fruit or a vegetable, 2) offered fruit but no vegetable, and 3) offered both a fruit and a vegetable. The number of sites offering a vegetable with no fruit was too few to use analytically.

### Data analysis

We used Stata/MP 14 statistical software (StataCorp) for simple descriptive counts, frequencies, cross-tabulation analyses, and regression modeling. We conducted two-sample *t* tests to identify descriptive characteristics associated with the dependent variables. Next, we developed 3 logistic regression models to identify characteristics independently associated with the dependent variables. We included independent variables in our models if *t* tests indicated statistically significant associations with the dependent variables or if there was a compelling theoretical reason in the absence of statistical associations (eg, participated in NSLP, whether the respondent was aware of the NAA HE standards).

## Results

We begin by providing characteristics of the sample and results of bivariate analyses. We then provide regression results.

### Sample characteristics

All analyses used the analytic sample (N = 684) (Table 1). Twenty-six percent of respondents reported they were at sites that received funding through the 21st CCLC program, 61% were at licensed sites, and 16% were at accredited sites. With respect to federal child nutrition programs, 28% of respondents reported their sites participated in CACFP and 26% participated in NSLP.

**Table 1:** Site, Program, and Respondent Characteristics Reported by Respondents to Afterschool Healthy Eating Survey, Fall 2015 (N = 684)

Characteristics	Survey Response	n (%)
Site is a 21st CCLC	Yes	175 (26)
	No	421 (62)
	Don’t know	88 (12)
Site is licensed	Yes	417 (61)
	No	208 (30)
	Don’t know	59 (9)
Site is accredited	Yes	112 (16)
	No	396 (58)
	Don’t know	176 (26)
Site participates in CACFP^a^	Yes	177 (28)
	No	297 (47)
	Don’t know	153 (25)
Site participates in NSLP^a^	Yes	162 (26)
	No	362 (58)
	Don’t know	103 (16)
Primary organizational affiliation of program	School district	242 (35)
	Miscellaneous public/private nonprofit	189 (28)
	Other small nonprofit/ for profit	190 (28)
	No affiliation	63 (9)
Respondent is NAA member	NAA member	422 (62)
	Not NAA member	262 (38)

Abbreviations: 21st CCLC, 21st Century Community Learning Center; CACFP, Child and Adult Care Food Program; NAA, National AfterSchool Association; NSLP, National School Lunch Program.

a Responses to CACFP and NSLP sum to less than 684 because of nonresponses; percentages reflect actual denominator.

Sites operated by a school or school district formed the largest category of site (35%). Another 28% were affiliated with nonprofit organizations such as YMCAs (7%), faith-based settings (6%), and Boys & Girls Clubs (5%) and an additional 28% of respondents reported affiliation with other small nonprofit and occasionally for-profit providers. Finally, 9% were independent providers reporting no organizational affiliation.

NAA members comprised 62% of the sample. We expected most respondents to be members because of our sampling strategy, though we also assumed some invitees would seek a substitute respondent.

### Descriptive and bivariate fruit and vegetable outcomes

Most respondents (63%; n = 433) reported offering a fruit, vegetables, or both as part of the snack served on the previous program day; these responses ranged from 63% among 21st CCLC sites to 75% among CACFP participants. Forty-two percent of sites offered fruit without a vegetable, 18% offered both a fruit and vegetable, and 3% offered vegetables only (Table 2).

**Table 2:** Fruit and Vegetables Offered with Afternoon Snack on Previous Program Day by Site Characteristics, Afterschool Healthy Eating Survey, Fall 2015

Characteristic	Stratum	Fruit, Vegetables, or Both	Only Fruit	Only Vegetables	Fruit and Vegetables	No Fruit or Vegetables
	n	n (%)	n (%)	n (%)	n (%)	n (%)
21st CCLC site^a^	175	111 (63)	68 (39)	4 (2)	39 (22)	64 (37)
Licensed site^a^	417	285 (68)	188 (45)	17 (4)	80 (19)	132 (32)
Accredited site^a^	112	77 (69)	46 (41)	2 (2)	29 (26)	35 (31)
Respondent is NAA member	422	275 (65)	203 (48)	11 (3)	61 (14)	147 (35)
Previously aware of NAA HE standards	395	260 (66)	171 (43)	12 (3)	77 (19)	135 (34)
Familiar with HE standards	187	130 (70)	93 (50)	5 (3)	32 (17)	57 (30)
Uses 1 or more of HE standards	390	276 (71)	179 (46)	12 (3)	85 (22)	114 (29)
Site participates in CACFP	177	132 (75)	84 (47)	6 (3)	42 (24)	45 (25)
Site participates in NSLP	162	103 (64)	72 (44)	2 (1)	29 (18)	59 (36)
Staff have healthy eating training 1x/y	228	160 (70)	100 (44)	11 (5)	49 (21)	68 (30)
Staff have healthy eating training > 1x/y	80	68 (85)	46 (58)	0 (0)	22 (28)	12 (15)
All programs	684	433 (63)	287 (42)	23 (3)	123 (18)	251 (37)

Abbreviations: 21st CCLC, 21st Century Community Learning Center; CACFP, Child and Adult Care Food Program; HE, healthy eating; NAA, National AfterSchool Association; NSLP, National School Lunch Program.

a Sites can belong to more than one characteristic category.

More than half of respondents (58%; n = 395) reported prior awareness that NAA had healthy eating standards, and 57% reported their sites were currently using one or more of the standards “to guide how [they] plan and serve food and/or beverages.”

Among 410 respondents that reported offering fruit, 79% reported it was fresh and 25% reported it was frozen, canned, or dried without added sugar. Among 146 respondents that reported serving vegetables, 88% reported vegetables were fresh, 18% offered vegetables frozen without added ingredients, and 20% offered vegetables canned with only water added. Almost 50% reported serving green vegetables, and 74% reported offering red, yellow, or orange vegetables. Respondents could select multiple quality responses to accommodate multi-item snacks.

In bivariate analyses, independent variables for school district, licensing, accreditation, NAA membership, CACFP, use of the NAA standards, and staff training were significantly associated with one or more of the outcome variables with an association of *P* < .05 or stronger. Offering fruit on the previous day was associated with school district affiliation, being a licensed or accredited site, NAA membership, participating in CACFP, using the NAA standards, and training staff more than once a year. Offering both fruit and vegetables was associated with school district affiliation, NAA membership, using the NAA standards, and training staff more than once a year. Offering fruit, vegetables, or both was associated with training staff more than once a year. Overall, 50% of sites provided healthy eating training to staff *not* involved in menu planning at least once a year, ranging from 40% of school district–affiliated sites to 72% of sites participating in CACFP (not shown).

### Regression results

We evaluated 3 binary (0/1) outcome measures: 1) offered a fruit or vegetable (n = 433), 2) offered fruit but no vegetable (n = 287), and 3) offered both a fruit and a vegetable (n = 123).

The odds of sites offering both a fruit and vegetable with snack when respondents were NAA members increased by a factor of 2 or more. Although NAA membership was a negative predictor of offering fruit alone, this was likely due to the strong association of sites offering a fruit and a vegetable together (Table 3).

**Table 3:** Independent Predictors of Sites Offering a Fruit, Vegetable, or Both with Snack on the Previous Day, Afterschool Healthy Eating Survey, Fall 2015 (N = 684)

Characteristic	Offered Fruit, Vegetables, or Both OR (SE), n = 433	*P* value	Offered Only Fruit OR (SE), n = 287	*P* value	Offered Fruit and Vegetable OR (SE), n = 123	*P* value
School district (1 = yes)	0.63 (0.11)	.01^a^	0.79 (0.14)	.19	0.66 (0.16)	.08
Licensed (1 = yes)	1.53 (0.27)	.02^a^	1.41 (0.25)	.05^a^	1.00 (0.23)	.99
Accredited (1 = yes)	1.01 (0.25)	.96	0.69 (0.16)	.10	2.01 (0.54)	.01^b^
Staff Trained in Healthy Eating						
Once a year	1.47 (0.29)	.05^a^	1.04 (0.19)	.83	1.61 (0.39)	.05^a^
More than once a year	3.52 (1.22)	<.001^b^	1.80 (0.48)	.03^a^	2.09 (0.67)	.02^a^
NAA member (1 = yes)	0.94 (0.16)	.39	0.52 (0.09)	<.001^b^	2.38 (0.52)	<.001^b^
Aware of NAA standards (1 = yes)	0.86 (0.16)	.39	0.91 (0.16)	.58	0.96 (0.22)	.87
CACFP (1 = yes)	1.37 (0.29)	.13	1.09 (0.21)	.66	1.30 (0.30)	.26
NSLP (1 = yes)	1.09 (0.22)	.65	1.30 (0.25)	.18	0.95 (0.24)	.84
Use NAA standards (1 = yes)	1.95 (0.36)	<.001^b^	1.42 (0.25)	.05^a^	1.70 (0.40)	.03^a^

Abbreviations: CACFP, Child and Adult Care Food Program; N/A, variable was not included in the model; NAA, National AfterSchool Association; NSLP, National School Lunch Program; OR, odds ratio; SE, standard error.

a *P* < .05.

b *P* < .01. Determined from logit models. Reference category for Staff Trained in Healthy Eating is “Never/less than once a year.”

Using the NAA standards for menu planning increased the probability of offering either a fruit or a vegetable, offering only fruit, and offering both a fruit and vegetable by a factor of 1.42 to 1.95. Similarly, training staff in healthy eating more than once a year, consistent with and exceeding NAA recommendations for at least once a year, increased by a factor of 1.80 to 3.52 the odds that the program provided either a fruit or a vegetable, fruit only, or both fruit and vegetables.

## Discussion

Although there is no national set of standards for food served in afterschool programs, many programs have adopted the NAA healthy eating standards in whole or in part. These standards include recommendations to offer fruit and vegetables daily. The 2015 NAA survey of afterschool snack quality among mostly NAA members found that most sites (63%) offered fruit, vegetables, or both during snack on the previous day, and they were most likely to offer fruit, either alone (42%) or with a vegetable (18%). They were unlikely to offer vegetables without also offering fruit (3%). In regression analyses, NAA-related predictors had the most consistent associations with positive outcomes. Controlling for other variables, we found that NAA members were more likely to offer vegetables alone or with fruit during snack and less likely to offer fruit alone. Respondents from sites that trained their staff in healthy eating more than once per year, thereby meeting and exceeding the NAA standards’ minimum of once per year, were more likely to offer fruit alone or with vegetables during snack. Moreover, the quality of the items offered showed that respondents preferentially selected the healthiest options, such as fresh fruit and vegetables. Substantial room for improvement was also evident: 37% of respondents did not offer fruit or vegetables with their previous snack.

Little national data exist on the presence of fruit and vegetables among snacks offered in afterschool programs. Others have studied afterschool snacks provided by the NSLP and found that few menus included fruit and vegetables (10). The data from this study indicate that the NAA HE standards are a meaningful component of efforts to improve nutrition in afterschool programs. Improving afterschool snack quality depends on a variety of factors, however. Although standards and regulations are important, implementation strategies aimed at improving program-level practices and staff-level capacity, including knowledge and skills, should be emphasized (13). Emerging literature indicates that capacity-building interventions have increased fruit and vegetable offerings at YMCA afterschool programs (11,12), though published research on interventions outside the YMCA setting and literature on the impact of the NAA standards is still lacking.

There are potential limitations to this study’s internal and external validity. Our purpose was to create a national snapshot using NAA’s membership database in the absence of a comprehensive national database of afterschool programs. Although we obtained a robust number of responses, and although they came from 49 states, we caution against generalizing results to all NAA members or afterschool programs for several reasons: our response rate was under 25%, sampling frames of US afterschool programs that would help assess broader generalizability do not exist, and we did not know the distribution of NAA members or NAA-engaged staff members among US afterschool programs. NAA staff members estimate that about 13% of afterschool professionals engage with the organization through membership, use of social media, or professional development (National AfterSchool Association, oral communication, November 2017). Although the survey instrument was cognitively tested, we did not validate the self-reported information, and reporting biases related to social desirability cannot be ruled out. Respondents also may have differed from nonrespondents, such as in their level of interest in healthy eating efforts. We also may have underestimated participation in CACFP and licensing and accreditation, given that “don’t know” responses for these variables ranged from 20% to 25%. Indeed, bivariate associations with these variables did not hold up in multivariate models. Given these caveats, we caution against using data from this survey to estimate afterschool participation rates in licensing, accreditation, CACFP, and NSLP. Finally, because the survey was cross-sectional, we cannot conclude that significant associations were causal.

This study offers initial insights into the impact of the NAA HEPA standards on afterschool snack quality and can help benchmark future assessments. Future research could assess nutrition in afterschool programs through record review (menus) and direct observation in a range of afterschool settings. Methodologic research could assess the validity of survey data on menu quality and build capacity for developing a more representative national sample of afterschool programs. In addition, qualitative research with stakeholders could assess barriers to providing fruit and vegetables, be they related to procurement, policy, or both. Despite its limitations, this study is unique in providing multistate data on programs affiliated with a range of organizations. We conclude that the NAA HE standards are associated with offering fruit and vegetables at afterschool programs affiliated with NAA members throughout the United States.
